# Authorship: attitudes and practice among Norwegian researchers

**DOI:** 10.1186/1472-6939-15-53

**Published:** 2014-07-02

**Authors:** Magne Nylenna, Frode Fagerbakk, Peter Kierulf

**Affiliations:** 1 The Norwegian Knowledge Centre for the Health Services, St. Olavs plass, P.O.Box 7004, N-0130 Oslo, Norway; 2 Faculty of Medicine, University of Oslo, St. Olavs plass, P.O.Box 7004, N-0130 Oslo, Norway; 3 Oslo University Hospital, St. Olavs plass, P.O.Box 7004, N-0130 Oslo, Norway

**Keywords:** Research, Publishing, Authorship, Misconduct, Dishonesty

## Abstract

**Background:**

Attitudes to, and practices of, scientific authorship vary. We have studied this variation among researchers in a university hospital and medical school in Norway.

**Methods:**

We invited all faculty, researchers and PhD students at Oslo University Hospital and the Faculty of Medicine, University of Oslo (approximately 2700) by e-mail to answer a web-based questionnaire in January 2013. We asked the researchers to report their authorship experiences and to score their agreement with, and ability to practice according to, 13 statements on authorship qualifications and criteria on a five-point Likert scale (1 = completely agree, 5 = completely disagree). The statements were taken from the International Committee of Medical Journal Editors (ICMJE) and other recommendations on authorship.

**Results:**

654 questionnaires were returned (response rate 24%); 25% of the respondents had published less than five scientific articles, 43% five to 49, and 32% more than 50 articles. 97% reported knowledge of defined authorship criteria, and 68% regarded breaches of these as scientific misconduct. 36% had experienced pressure to include undeserved authors in their papers, more in basic science (46%) than in community medicine (25%). 29% reported that they had been denied authorship they believed they deserved. Researchers with less than six years of research experience found authorship decisions more difficult than more experienced researchers (48% vs 30%).

The respondents’ agreement with the statements on authorship was higher than their self-reported ability to follow them for all statements. Average scores for agreement and practice for all statements combined were 1.4 vs 2.3. The discrepancy between attitude and practice declined with publishing experience. For the core ICMJE authorship requirements the average difference between attitude and practice was 1.2 among those who had published less than 5 articles and 0.7 among those who had published 50 articles or more (p < 0.05).

**Conclusions:**

Almost all the responding researchers had knowledge of formal authorship requirements. Most of them agreed with the criteria, but found it harder to put them into practice. More experienced researchers found decisions on authorship and about the order of authors easier than less experienced researchers.

## Background

Authorship is a constant topic for discussion in scientific publishing. A systematic review of 123 articles about authorship issues revealed a wide variety of problems, the most frequent being authorship perceptions, definitions and practices [1]. Problems about the threshold of involvement qualifying for authorship as well as the order of listing authors are unresolved [2]. Criteria have been developed by the International Committee of Medical Journal Editors (ICMJE) to establish standardized definitions and thereby secure responsibility and accountability of authorship in medical journals [3]. Hundreds of journals have adopted these criteria, but controversies still exist and breaches of authorship guidelines are common. In discussions on responsible conduct of research, it has been said that “no issue is more ubiquitous and contentious than the question of authorship” [4]. Inappropriate authorship occurs frequently. A meta-analysis of 14 surveys showed that an average of 29% of researchers had experienced misuse of authorship [1]. In a study of six high impact medical journals in 2008 the prevalence of so-called honorary authorship was 25% in original research papers [5].

Disputes on authorship used to be the most frequent reason for investigating alleged cases of scientific misconduct in the Nordic countries [6]. A recent survey of doctoral students in Norway showed that 11% had experienced unethical pressure concerning the order of authors during the last 12 months [7]. As lecturers in research ethics and administrators of research institutions, we have experienced several authorship disputes. However, our knowledge of attitudes to, and practices of, authorship in different groups of researchers is limited and to explore this we have done a survey among Norwegian medical researchers.

## Methods

We conducted an anonymous web-based survey (in Norwegian) among researchers in Oslo, Norway between the 3^rd^ and 11^th^ of January 2013. We invited all faculty, researchers and PhD students at Oslo University Hospital and the Medical Faculty, University of Oslo (approximately 2700) by e-mail to answer a web-based questionnaire. The survey was administered using the data collection tool “Nettskjema” [8]. We informed the researchers about the study through an open invitation and they consented to their participation by answering the questionnaire.

We measured respondents’ experience by the number of years they had conducted research and the number of papers they had published. Participants viewed 13 statements on authorship qualifications and criteria, one at a time, and were asked to rate two items for each statement: 1) their level of agreement with the authorship principle and 2) their perceived ability to put that principle into practice. We used statements from the International Committee of Medical Journal Editors (ICMJE) [3] and other national and international recommendations on authorship (Table 1). Statements 1–4 were the core requirements for authorship according to the ICMJE as of January 2013 [3]. The statements included in the questionnaire were agreed upon by consensus of the investigators. Ratings were based on five-point Likert scale (ranging from 1 = completely agree to 5 = completely disagree). The questionnaire was piloted and tested among researchers at the Norwegian Knowledge Centre for Health Services during the fall of 2012.

**Table 1:** Respondents’ judgement of the statements presented to them, scored on a five-level Likert scale (1 = completely agree, 5 = completely disagree) (N = 654)

**Statement**	**Mean**	**(95% CI)**
1 An author must take responsibility for at least one component of the work, should be able to identify who is responsible for each other component, and should ideally be confident in their co-authors’ ability and integrity	1.59	(1.53 – 1.65)
Easy to practice this principle	2.84	(2.76 – 2.92)
2 Authors should have given substantial contributions to conception and design, acquisition of data, or analysis and interpretation of data	1.25	(1.20 – 1.29)
Easy to practice this principle	2.52	(2.44 – 2.60)
3 Authors should have drafted the article or revised it critically for important intellectual content	1.63	(1.55 – 1.70)
Easy to practice this principle	2.48	(2.39 – 2.56)
4 Authors should have given final approval of the version to be published	1.08	(1.05 – 1.11)
Easy to practice this principle	1.64	(1.57 – 1.71)
5 For collective authorship, e.g. group or organization, one or more individuals responsible should be named	1.38	(1.31 – 1.45)
Easy to practice this principle	1.92	(1.82 – 2.02)
6 Contributors who do not meet the criteria for authorship should be listed in an acknowledgments section. The contribution of each should be specified.	1.27	(1.22 – 1.32)
Easy to practice this principle	1.90	(1.82 – 1.98)
7 The person who has contributed most to the work should be first author	1.29	(1.24 – 1.34)
Easy to practice this principle	2.00	(1.92 – 2.07)
8 The last author should normally be the principal supervisor	1.33	(1.27 – 1.39)
Easy to practice this principle	2.02	(1.93 – 2.10)
9 Authors should be named in the order they have contributed to the work (most contributions first) with a possible exception for the last author	1.63	(1.55 – 1.71)
Easy to practice this principle	2.55	(2.47 – 2.63)
10 Authors should decide among themselves who qualifies for authorship and the order of authors before submitting a manuscript	1.24	(1.19 – 1.29)
Easy to practice this principle	2.16	(2.08 – 2.25)
11 Acquisition of funding alone does not constitute authorship	1.24	(1.19 – 1.29)
Easy to practice this principle	2.02	(1.92 – 2.12)
12 General supervision of the research group alone does not constitute authorship	1.71	(1.64 – 1.79)
Easy to practice this principle	2.66	(2.56 – 2.76)
13 An author is normally someone who has given substantial, intellectual contributions to a published study	1.51	(1.44 – 1.57)
Easy to practice this principle	2.43	(2.35 – 2.51)

We calculated mean scores for each individual rating and for pairs of ratings combined and carried out simple statistical analyses (t-tests, 95% confidence intervals (CI)).

According to the Norwegian Health Research Act, surveys for studying attitudes and practices among researchers fall outside the remit of the Research Ethics Committees. Therefore this project did not require approval from a Research Ethics Committee.

## Results

654 researchers answered the questionnaire, 310 females (47%) and 344 males (53%). 182 (28%) were under 40 years, 339 (52%) between 40 and 60 years and 133 (20%) over the age of 60. 234 (36%) had five or fewer years’ experience as researchers and 420 (64%) had six or more years. 164 (25%) had been first author or co-author of fewer than five scientific papers, 282 (43%) had published between five and 49 papers, and 208 (32%) had published 50 papers or more. 554 (90%) of the respondents held full- or part-time positions related to the Medical Faculty, University of Oslo; 326 (50%) at the Institute og Clinical Medicine, 87 (13%) at the Institute of Basic Medical Sciences, 84 (13%) at the Institute of Health and Society. 57 (9%) did not specify their institutional relationship, while 100 (15%) had no formal relations to the Medical Faculty.

634 (97%) of the respondents had knowledge of existing criteria and guidelines for authorship. 445 (68%) regarded breaches of authorship guidelines as scientific misconduct (Table 2). Significantly more men than women disagreed with the statement that breaches of authorship guidelines were scientific misconduct (14% vs 5% respectively, p < 0.01). Researchers with fewer than five published articles were more likely to regard breaches as misconduct than researchers with 50 or more publications (73 vs 63%, p < 0.05).

**Table 2:** Responses to the question “Do you think breaches of authorship guidelines should be called scientific misconduct?”

	**Yes (%)**	**No (%)**	**Don’t know (%)**
Females N = 310	217 (70%)	15 (5%)	78 (25%)
Males N = 344	228 (66%)	48 (14%)	68 (20%)
Total N = 654	445 (68%)	63 (10%)	146 (22%)

Reported experiences with authorship disputes and problems were closely related to the number of papers published by respondents (Table 3). 377 (58%) had been involved in situations with disagreement about authorship. 237 (36%) reported that they find decisions on authorship and the order of authors in general difficult, 108 (31%) of males and 129 (42%) of females (p < 0,05). 191 (29%) reported that they had been excluded from authorship when they believed they deserved it, 117 (34%) among males and 75 (24%) among females (p < 0,05). 233 (36%) had experienced pressure to include undeserving (i.e. ‘guest’ or ‘honorary’) authors in their articles, more in basic science (46%) than in community medicine (25%). Researchers with less than six years of research experience found decisions on authorship in general more difficult than more experienced researchers (48% vs 30%, p < 0,01).

**Table 3:** Respondents’ reporting on authorship experience according to own number of published papers (N = 654)

**Publication experience**	**Have been involved in situations with disagreement on authorship (%)**	**Have been forced to accept non-deserved coauthors (%)**	**Have (in own opinion) been excluded from deserved authorship (%)**	**Finding decision on authorship and order of authors in general difficult (%)**
< 5 publications N = 164	64 (39%)	47 (29%)	13 (8%)	86 (52%)
5-49 publications N = 282	175 (62%)	116 (41%)	83 (29%)	116 (41%)
> 50 publications N = 208	138 (66%)	70 (34%)	95 (46%)	35 (17%)

The respondents’ judgments of the statements on authorship is shown in Table 1. Their level of agreement was higher than their reported ability to put the principles into practice for all statements. For all 13 statements combined the average score for agreement (attitude) was 1.39 (95% CI 1.37-1.42) versus 2.25 (2.20-2.30) for the judgment of how easy it is to practice according to the statement (practice). The discrepancy between attitude and practice was highest for the two ICMJE requirements [3]: “*An author must take responsibility for at least one component of the work, should be able to identify who is responsible for each other component, and should ideally be confident in their co-authors’ ability and integrity”* (statement 1) and *“Authorship credit should be based on substantial contributions to conception and design, acquisition of data, or analysis and interpretation of data”* (statement 2). The discrepancy between attitude and practice declined with publishing experience mainly because more experienced researchers found it easier to practice according to the principles. For the core ICMJE requirements (statements 1–4 combined) the average difference between attitude and practice was 1.2 among those who had published fewer than 5 articles and 0.7 among those who had published 50 articles or more (p < 0,05).

The lowest level of agreement was reported for the statement *“General supervision of the research group alone does not constitute authorship”* (statement 12).

## Discussion

Almost all respondents reported having knowledge of formal authorship criteria and two thirds considered breaches of such criteria to be scientific misconduct. Almost two out of five find decisions on authorship difficult in general, and higher proportions of women (than men) and less experienced (than more experienced) researchers reported such problems. Almost one out of three believed that they had been excluded from deserved authorship and more than one third had experienced pressure to include undeserved authors in their papers. The level of agreement with the statements was high, but for all statements respondents found it harder to put them into practice than to agree with them. This discrepancy diminished with increasing publishing experience.

### Strengths and limitations

Web-based surveys are popular as they can reach many people at a low cost. Response rates are generally low when a wide range of people is electronically invited to participate. When all potential respondents are taken into account the response rate in our study was low, only 24%. Web surveys have lower response rates than traditional mail surveys. This has been confirmed by a meta-analysis of studies comparing mail-surveys and web-surveys in which 16 of the 39 studies (44%) had a lower response rate than our 24% [9].The relationship between response rate and no-response bias is also limited for this kind of survey [10]. However, our respondents represented a wide variety of researchers according to age, gender, experience and field of research and 654 respondents gives a good basis for studying self-reported attitude and practice.

The part of our survey based on statements from The International Committee of Medical Journal Editors referred to recommendations in the Uniform Requirements for Manuscripts Submitted to Biomedical Journals as of January 2013. These recommendations were revised in August 2013 [11]. The most important change was the addition of a new criterion for authorship to emphasize each author’s responsibility to stand by the integrity of the entire work and reads: “Agreement to be accountable for all aspects of the work in ensuring that questions related to the accuracy or integrity of any part of the work are appropriately investigated and resolved.” [3]. The recommendations were at the same time renamed “Recommendations for the Conduct, Reporting, Editing, and Publication of Scholarly work in Medical Journals” [3].

### Interpretations

Our findings show that authorship is a problem among medical researchers. Despite the fact that researchers were familiar with formal authorship requirements, and even regarded breaches of these as ethically unacceptable, a high proportion had experienced such breaches. A majority of researchers have been or will be involved in disagreements on authorship. The more papers are published, the more opportunities arise for authorship disputes.

More respondents in our study than in similar studies were aware of existing authorship criteria [12-15]. Newer studies report a higher level of such awareness than older studies, indicating that knowledge of formal authorship criteria among researchers has increased over the last 20 years [12-15]. More respondents in our study than among Indian medical researchers in 2006 [13] and American plastic surgeons in 2003 and 2011 [14] had been involved in disputes over authorship. The reported frequency of self-experienced authorship abuse among our respondents was in line with other findings [1,14].

It is well known that doctors’ behaviour does not always reflect the principles and standards they agree to [16]. Our study reveals that this also applies to scientific authorship.

The diminishing gap between attitudes and practice with increasing publishing experience gives reason for reflections. We found that more experienced researchers find it easier to put authorship recommendations into practice. Whether this is due to a more liberal approach to authorship is unknown. Possibly, more experienced researchers have come to the understanding that rules or guidelines, such as those proclaimed by The World Association of Medical Editors (WAME) and International Committee of Medical Journal Editors (ICMJE) actually should serve as “guidelines” and “recommendations” rather than “strict rules”. If this is the case, it may raise the question as to how these researchers *actually practice* authorship. Awareness and understanding of the recommended rules obviously is “a must”, nevertheless many researchers find the ICMJE guidelines too restrictive [17]. Furthermore, modern scientific research increasingly depends on advanced technologies fostering large scale and multi-disciplinary teams. Thus, researchers are frequently challenged as to guidelines and practice.

To what extent a complete disclosure statement, including not only all potential or actual conflicts of interest but also detailed information on individual contributions to the study and publication, will increase discrepancies between guidelines and practice remains to be seen. Although a complete disclosure statement may appear ideal, it may take years of education to improve authorship practices. In this connection it should be pointed out that byline authorship does not, in itself, communicate what each author actually has done. Many journals (such as *BMC Medical Ethics*) therefore ask authors to specify each person’s contributions [18]. Contributors to a study who do not meet all the criteria for authorship should not be listed as authors but, with written permission, should be acknowledged [3]. A proposal to completely replace the concept of authorship with specified contributions [19] did not succeed. To compensate for what has been seen as unfair credit given for co-authorship compared with single-authorship, it has recently been suggested to assign a numerical value to the degree of relative contribution, thus creating a contribution-specific index for each author and to use this when evaluating research productivity [20].

Ideally, authorship practice in a medical school, such as the Faculty of Medicine, University of Oslo, and in a large university hospital, such as the Oslo University Hospital should follow generally accepted international guidelines. This would imply that such guidelines are well known. As an initial “rule of thumb” it appears wise (and necessary) to determine authorship prior to, or at the start of project planning, “through an open dialogue with all individuals participating in the research” [21]. Such an approach would take into account “not only what decision is made but also how it is made” [21]. In light of the understanding of how dynamic the process of authorship in a research project actually is, and should be, such transparency could make it easier for the engaged researchers to finalize authorship.

### Consequences

Authorship criteria and requirements have been discussed and defined by several organizations and institutions. In spite of this, authorship is seen as an underserved area of education in the responsible conduct of research [4,22]. To avoid inappropriate authorship, all those involved, research institutions, authors, editors and publishers, must understand the importance of fair crediting and take measures to ensure adherence to current guidelines and requirements [2,23]. It is, however, discouraging that an intervention study among medical students found that instruction about formal authorship criteria had no effect on students’ deciding about authorship dilemmas [24].

Like other academic institutions The Faculty of Medicine, University of Oslo, as well as the Oslo University Hospital, face the challenges of informing their researchers about general authorship rules, and how to practice such rules. To reach these ends, policy statements should be posted on institutions’ websites. However, web-posting and instructions alone will never suffice. A cultural change is needed, since decisions on authorship seem to be more related to moral judgements than to awareness of formal criteria [24]. Interactive lessons, “live-practice”, short seminars and working groups detailing complex problems, and promoting Faculty and Hospital standards should be initiated.

## Conclusions

The respondents in this study were familiar with formal authorship requirements and even regarded breaches of these as ethically unacceptable. Nevertheless, a high proportion had experienced such breaches. In general, they found it much easier to agree with the formal criteria for authorship than to practice according to these principles.

We do not know whether the increased ease in practicing authorship principles reported by more experienced researchers is due to a more liberal approach to authorship than for those with less experience.

## Competing interests

The authors declare that they have no competing interests.

## Authors’ contributions

MN and PK planned, designed, and conducted the study. FF did the statistical analyses. All authors analyzed the findings. MN drafted the manuscript, PK and FF revised the text. All authors have given final approval of the paper.

## Pre-publication history

The pre-publication history for this paper can be accessed here:

http://www.biomedcentral.com/1472-6939/15/53/prepub

## References

[B1] Marušić A Bošnjak L Jerončić A A systematic review of research on the meaning, ethics and practices of authorship across scholarly disciplines PLoS One 2011 6 9 e23477 doi:10.1371/journal.pone.0023477 10.1371/journal.pone.0023477 21931600 PMC3169533

[B2] Wager E Recognition, reward and responsibility: why the authorship of scientific papers matter Maturitas 2009 62 109 12 doi:10.1016/j.maturitas.2008.12.001 10.1016/j.maturitas.2008.12.001 19147308

[B3] International Committee of Medical Journal Editors Defining the role of authors and contributors http://www.icmje.org/recommendations/browse/roles-and-responsibilities/defining-the-role-of-authors-and-contributors.html

[B4] Kalichman MW Overview: underserved areas of education in the responsible conduct of research: authorship Sci Eng Ethics 2011 17 335 9 doi:10.1007/s11948-011-9281-3 10.1007/s11948-011-9281-3 21611821 PMC3428198

[B5] Wislar JS Flanagin A Fontanarosa PB DeAngelis CD Honorary and ghost authorship in high impact biomedical journals: a cross sectional survey BMJ 2011 343 d6128 10.1136/bmj.d6128 22028479 PMC3202014

[B6] Nylenna M Andersen D Dahlquist G Sarvas M Aakvaag A Handling of scientific dishonesty in the Nordic countries Lancet 1999 354 57 61 10.1016/S0140-6736(98)07133-5 10406378

[B7] Hofmann B Myhr AI Holm S Scientific dishonesty – a nationwide survey of doctoral students in Norway BMC Med Ethics 2013 14 3 doi:10.1186/1472-6939-14-3 10.1186/1472-6939-14-3 23289954 PMC3545724

[B8] University of Oslo Nettskjema [Web-scheme] http://www.uio.no/english/services/it/adm-services/nettskjema/

[B9] Shih T-H Fan X Comparing response rates from web and mail surveys: a Meta-Analysis.Field Methods 2008 20 249

[B10] Keeter S Miller C Kohut A Groves RM Presser S Consequences of reducing nonresponse in a national telephone survey Public Opin Q 2000 64 125 148 doi:10.1086/317759 10.1086/317759 10984330

[B11] International Committee of Medical Journal Editors The New ICMJE Recommendations 2013 http://www.icmje.org/news-and-editorials/new_rec_aug2013.html 25017830

[B12] Bhopal R Rankin J McColl E Thomas L Kaner E Stacy R Pearson P Vernon B Rodgers H The vexed question of authorship: views of researchers in a British medical faculty BMJ 1997 314 1009 10.1136/bmj.314.7086.1009 9112845 PMC2126416

[B13] Dhaliwal U1 Singh N Bhatia A Awareness of authorship criteria and conflict: survey in a medical institution in India MedGenMed 2006 12 8 52 PMC1868341 17415332

[B14] Reinisch JF Li WY Yu DC Walker JW Authorship conflicts: a study of awareness of authorship criteria among academic plastic surgeons Plast Reconstr Surg 2013 132 303e 310e doi:10.1097/PRS.0b013e3182958b5a 10.1097/PRS.0b013e3182958b5a 23897358

[B15] Karani R Ognibene FP Fallar R Gliatto P Medical students’ experiences with authorship in biomedical research: a national survey Acad Med 2013 88 364 8 doi:10.1097/ACM.0b013e31827fc6ae 10.1097/ACM.0b013e31827fc6ae 23348080

[B16] Campbell EG Regan S Gruen RL Ferris TG Rao SR Cleary PD Blumenthal D Professionalism in medicine: results of a national survey of physicians Ann Intern Med 2007 147 795 802 10.7326/0003-4819-147-11-200712040-00012 18056665

[B17] Pignatelli B Maisonneuve H Chapuis F Authorship ignorance: views of researchers in French clinical settings J Med Ethics 2005 31 578 81 10.1136/jme.2004.009449 16199598 PMC1734035

[B18] Authors’ contributions http://www.biomedcentral.com/bmcmedethics/authors/instructions/researcharticle#formatting-contributions

[B19] Rennie D Yank V Emanuel L When authorship fails. A proposal to make contributors accountable JAMA 1997 278 579 85 10.1001/jama.1997.03550070071041 9268280

[B20] Kovacs J Honorary authorship epidemic in scholarly publications? How the current use of citation-based evaluative metrics make (pseudo) honorary authors from honest contributors of every multi-author article J Med Ethics 2013 39 509 12 doi:10.1136/medethics-2012-100568 10.1136/medethics-2012-100568 22865926

[B21] Smith E Williams-Jones B Authorship and responsibility in health sciences research: a review of procedures for fairly allocating authorship in multi-author studies Sci Eng Ethics 2012 18 199 212 doi:10.1007/s11948-011-9263-5 10.1007/s11948-011-9263-5 21312000

[B22] Macrina FL Teaching authorship and publication practices in the biomedical and life sciences Sci Eng Ethics 2011 17 341 54 10.1007/s11948-011-9275-1 21533836

[B23] Gasparyan AY Ayvazyan L Kitas GD Authorship problems in scholarly journals: considerations for authors, peer reviewers and editors Rheumatol Int 2013 33 277 84 10.1007/s00296-012-2582-2 23124697

[B24] Hren D Sambunjak D Marušić M Marušić A Medical students’ decisions about authorship in disputable situations: intervention study Sci Eng Ethics 2013 19 641 51 doi:10.1007/s11948-012-9358-7 10.1007/s11948-012-9358-7 22382923

